# A new genus of metalmark moths (Lepidoptera, Choreutidae) with Afrotropical and Australasian distribution

**DOI:** 10.3897/zookeys.355.6158

**Published:** 2013-11-25

**Authors:** Jadranka Rota, Scott E. Miller

**Affiliations:** 1 Laboratory of Genetics and Zoological Museum, Department of Biology, University of Turku, FI-20014 Turku, Finland; 2 National Museum of Natural History, Smithsonian Institution, P.O. Box 37012, Washington, D.C., 20013-7012, USA

**Keywords:** Alpha taxonomy, DNA barcoding, *Ficus* spp., Kenya, *Niveas agassizi*, *Niveas kone*, Papua New Guinea, Solomon Islands, phylogenetics

## Abstract

*Niveas* Rota, new genus, and its two new species, *N. agassizi* Rota, new species, and *N. kone* Rota, new species, are described and illustrated. *Niveas* is assigned to the subfamily Choreutinae based on morphological and molecular data. *Niveas agassizi* is currently known only from Kenya and only from female specimens. *Niveas kone* has been found on the Solomon Islands and in Papua New Guinea (PNG). In PNG, larvae of this species have been reared from several species of *Ficus* (Moraceae). The two species are superficially quite dissimilar from each other. However, they share features in wing pattern and venation, as well as female genitalia, and the molecular data strongly support the monophyly of *Niveas*.

## Introduction

Choreutidae, commonly known as metalmark moths, are a family of micro-moths with a worldwide distribution. The family is most species-rich in the tropics, and, as is the case for numerous other small tropical invertebrates, much of its richness is still unknown to science (unpublished data). Currently, 406 species of choreutids are described (Nieukerken et al. 2011).

Choreutids are medium-sized micro-moths with wingspans ranging from about one to two centimeters, often with bright colors and iridescent markings on their wings (Diakonoff 1986). They are diurnal with only some species attracted to lights at night (personal observation), making them a fairly rare group in museum collections. In our experience, large-scale rearing projects result in finding more species of choreutids than employing light traps.

Through exactly such efforts over the past 20 years in Papua New Guinea (PNG), the Binatang Research Center (BRC), with a large international group of collaborators focusing on the ecology of herbivorous insects and their host plants (Miller et al. 2003; Craft et al. 2010; Novotny et al. 2010; Hrcek et al. 2011; Hrcek et al. 2013; Miller et al. 2013), the number of known species of choreutids and our knowledge of their biology have greatly increased. One of the many new species of choreutids found in PNG during this project is sufficiently different from all described species that it requires a new genus.

Coincidentally, through separate collecting efforts by David Agassiz in Africa, a related species was discovered in Kenya. Herein these two species, as well as the genus to which they belong, are described and illustrated, and the phylogenetic position of the new genus within the family is discussed.

**Figures 1–4.**
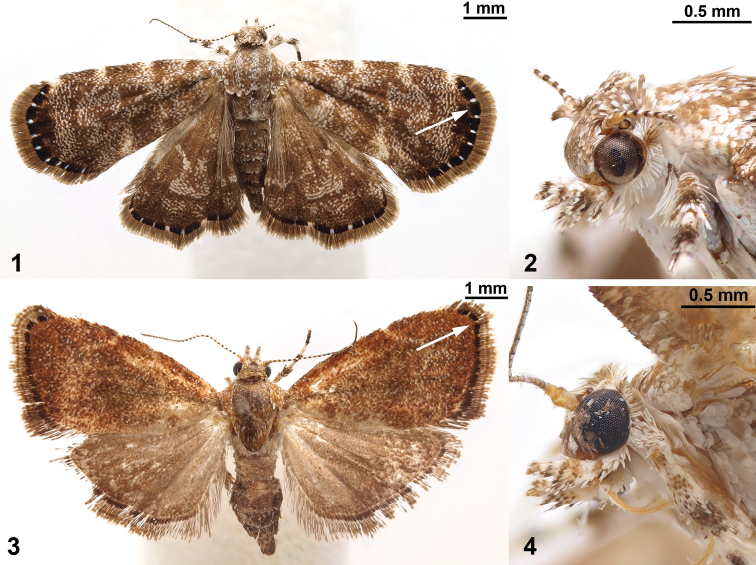
*Niveas kone*: **1** Habitus **2** Head. *Niveas agassizi*: **3** Habitus **4** Head. (In Figs 1 and 3 arrows point at the terminal black band enclosing white spots.)

The shared presence of the terminal black band with white spots in the forewing (arrows in Figs 1, 3) was the first indication that *Niveas kone* Rota, sp. n. and *Niveas agassizi* Rota, sp. n. might be related. Initially this relationship seemed unlikely because of the disjunct geographical distribution of the two (*Niveas kone* being distributed in the Australasian Region and *Niveas agassizi* in the Afrotropical Region) and because their DNA barcodes did not suggest a close relationship. However, once the similarities in wing venation and female genitalia were noticed, and we included nuclear genes in the analysis with a more extensive choreutid molecular dataset, the results strongly supported the close relationship between *Niveas kone* and *Niveas agassizi*.

## Methods

All material examined is listed in Table 1. Layered photographs of specimens and slides were taken using an Olympus SZX16 microscope with motorized focus drive attached to an Olympus E520 digital camera. The photographs were then combined by using the programs Deep Focus 3.1 and Quick Photo Camera 2.3. The wing venation drawing was made digitally in Adobe Illustrator CS3 overlaid on top of a slide photograph. All images were improved in Adobe Photoshop CS3. Genitalic dissections and terminology follow Rota (2008b).

**Table 1.** Material examined.

Species	Type	Country	Province	Locality	Date	Collector	ID number	Host plant	Slide number	GenBank
*Niveas kone*	Paratype	PNG	Madang	Baitabag Vill.	04/09/95	BRC	USNM ENT 730507	*Ficus nodosa*		
*Niveas kone*	Paratype	PNG	Madang	Baitabag Vill.	08/30/95	BRC	USNM ENT 730558	*Ficus nodosa*		
*Niveas kone*	Paratype	PNG	Madang	Baitabag Vill.	08/30/95	BRC	USNM ENT 730572	*Ficus nodosa*		HQ946542
*Niveas kone*	Paratype	PNG	Madang	Baitabag Vill.	06/16/95	BRC	USNM ENT 730508	*Ficus variegata*		
*Niveas kone*	Paratype	PNG	Madang	Baitabag Vill.	03/19/96	BRC	USNM ENT 730513	*Ficus variegata*		HQ946551
*Niveas kone*	Paratype	PNG	Madang	Baitabag Vill.	04/09/95	BRC	USNM ENT 730529	*Ficus variegata*		KF714836
*Niveas kone*	Paratype	PNG	Madang	Baitabag Vill.	03/19/96	BRC	USNM ENT 730543	*Ficus variegata*		
*Niveas kone*	Paratype	PNG	Madang	Baitabag Vill.	03/19/96	BRC	USNM ENT 730551	*Ficus variegata*		
*Niveas kone*	Paratype	PNG	Madang	Kamba (Mis)	10/20/95	BRC	USNM ENT 730576	*Ficus variegata*		HQ946555
*Niveas kone*	Paratype	PNG	Madang	Malapau (Riwo)	03/20/95	BRC	USNM ENT 730498	*Ficus variegata*		HQ946554
*Niveas kone*	Paratype	PNG	Madang	Malapau (Riwo)	03/20/95	BRC	USNM ENT 730519	*Ficus variegata*		HQ946553
*Niveas kone*	Paratype	PNG	Madang	Malapau (Riwo)	03/20/95	BRC	USNM ENT 730535	*Ficus variegata*		HQ946552
*Niveas kone*	Paratype	PNG	Madang	Mililat (Riwo)	05/22/95	BRC	USNM ENT 730604	*Ficus nodosa*		HQ946544
*Niveas kone*	Paratype	PNG	Madang	Mis Vill.	03/20/96	BRC	USNM ENT 730528	*Ficus nodosa*		HQ946543
*Niveas kone*	Paratype	PNG	Madang	Ohu Vill.	04/09/95	BRC	USNM ENT 730560	*Ficus botryocarpa*		HQ946538
*Niveas kone*	Paratype	PNG	Madang	Ohu Vill.	05/09/95	BRC	USNM ENT 730602	*Ficus botryocarpa*		HQ946539
*Niveas kone*	Paratype	PNG	Madang	Ohu Vill.	12/01/96	BRC	USNM ENT 730542	*Ficus phaeosyce*		KF714835
*Niveas kone*	Paratype	PNG	Madang	Ohu Vill.	12/02/94	BRC	USNM ENT 730502	*Ficus pungens*		HQ946546
*Niveas kone*	Paratype	PNG	Madang	Ohu Vill.	12/09/95	BRC	USNM ENT 730518	*Ficus variegata*	female genitalia 92352	HQ946549
*Niveas kone*	Paratype	PNG	Madang	Ohu Vill.	03/16/95	BRC	USNM ENT 730509	*Ficus variegata*	male genitalia 92355	HQ946550
*Niveas kone*	Paratype	PNG	Madang	Ohu Vill.	03/16/95	BRC	USNM ENT 730492	*Ficus variegata*		
*Niveas kone*	Paratype	PNG	Madang	Ohu Vill.	03/25/96	BRC	USNM ENT 730493	*Ficus variegata*		
*Niveas kone*	Paratype	PNG	Madang	Ohu Vill.	05/09/95	BRC	USNM ENT 730500	*Ficus variegata*		
*Niveas kone*	Paratype	PNG	Madang	Ohu Vill.	03/22/95	BRC	USNM ENT 730504	*Ficus variegata*		
*Niveas kone*	Paratype	PNG	Madang	Ohu Vill.	12/13/94	BRC	USNM ENT 730510	*Ficus variegata*		
*Niveas kone*	Holotype	PNG	Madang	Ohu Vill.	03/13/95	BRC	USNM ENT 730516	*Ficus variegata*		HQ946548
*Niveas kone*	Paratype	PNG	Madang	Ohu Vill.	08/09/95	BRC	USNM ENT 730517	*Ficus variegata*		
*Niveas kone*	Paratype	PNG	Madang	Ohu Vill.	03/16/95	BRC	USNM ENT 730520	*Ficus variegata*		
*Niveas kone*	Paratype	PNG	Madang	Ohu Vill.	05/26/95	BRC	USNM ENT 730521	*Ficus variegata*		
*Niveas kone*	Paratype	PNG	Madang	Ohu Vill.	05/09/95	BRC	USNM ENT 730522	*Ficus variegata*		
*Niveas kone*	Paratype	PNG	Madang	Ohu Vill.	03/29/95	BRC	USNM ENT 730523	*Ficus variegata*		
*Niveas kone*	Paratype	PNG	Madang	Ohu Vill.	06/16/95	BRC	USNM ENT 730524	*Ficus variegata*		
*Niveas kone*	Paratype	PNG	Madang	Ohu Vill.	05/11/96	BRC	USNM ENT 730525	*Ficus variegata*		
*Niveas kone*	Paratype	PNG	Madang	Ohu Vill.	06/27/95	BRC	USNM ENT 730526	*Ficus variegata*		
*Niveas kone*	Paratype	PNG	Madang	Ohu Vill.	06/16/95	BRC	USNM ENT 730531	*Ficus variegata*		
*Niveas kone*	Paratype	PNG	Madang	Ohu Vill.	12/13/94	BRC	USNM ENT 730533	*Ficus variegata*		
*Niveas kone*	Paratype	PNG	Madang	Ohu Vill.	05/09/95	BRC	USNM ENT 730553	*Ficus variegata*		
*Niveas kone*	Paratype	PNG	Madang	Ohu Vill.	12/09/95	BRC	USNM ENT 730564	*Ficus variegata*		
*Niveas kone*	Paratype	PNG	Madang	Ohu Vill.	05/09/95	BRC	USNM ENT 730588	*Ficus variegata*		
*Niveas kone*	Paratype	PNG	Madang	Ohu Vill.	05/09/95	BRC	USNM ENT 730595	*Ficus variegata*		
*Niveas kone*	Paratype	PNG	Madang	Ohu Vill.	09/10/95	BRC	USNM ENT 730565	*Ficus wassa*		HQ946545
*Niveas kone*	Paratype	PNG	Madang	Pau Vill.	12/13/95	BRC	USNM ENT 730515	*Ficus variegata*		KF714837
*Niveas kone*	Paratype	PNG	Madang	Pau Vill.	12/13/95	BRC	USNM ENT 730547	*Ficus variegata*		KF714833
*Niveas kone*	Paratype	PNG	Madang	Reinduk	03/28/95	BRC	USNM ENT 730527	*Ficus variegata*		KF714834
*Niveas kone*	Paratype	PNG	Madang	Tab Is	01/31/95	BRC	USNM ENT 730506	*Ficus nodosa*		
*Niveas kone*	Paratype	PNG	Madang	Tab Is	01/31/95	BRC	USNM ENT 730514	*Ficus nodosa*		KF714832
*Niveas kone*	Paratype	PNG	Madang	Tab Is	01/31/95	BRC	USNM ENT 730532	*Ficus nodosa*		HQ946540
*Niveas kone*	Paratype	PNG	Madang	Tab Is	01/31/95	BRC	USNM ENT 730538	*Ficus nodosa*		HQ946541
*Niveas kone*	Paratype	PNG	Madang	Wanang Vill.	07/31/07	BRC	USNM ENT 660733	*Ficus variegata*		
*Niveas kone*	Paratype	PNG	Madang	Wanang Vill.	11/05/07	BRC	USNM ENT 660794	*Ficus variegata*		
*Niveas kone*	Paratype	PNG	Madang	Wanang Vill.	02/21/06	BRC	USNM ENT 660722	*unknown*		HQ946547
*Niveas kone*	Paratype	Solomon Is.	Guadalcanal	Roroni, 35 km E of Honiara; 10 m	05/13/64	R. Straatman	unassigned	*unknown*	wing 137601; female genitalia 137600	
*Niveas kone*	Paratype	Solomon Is.	Guadalcanal	Roroni, 35 km E of Honiara; 10 m	05/13/64	R. Straatman	unassigned	*unknown*		
*Niveas kone*	Paratype	Solomon Is.	Guadalcanal	Nini Ck., 35 km SE of Honiara	08/05/64	R. Straatman	unassigned	*unknown*		
*Niveas agassizi*	Paratype	Kenya	County of Kwale	Mwabungu	08/19/00	David Agassiz	USNM ENT 730794	*unknown*		HQ946715
*Niveas agassizi*	Holotype	Kenya	County of Kwale	Mwabungu	08/19/00	David Agassiz	USNM ENT 730793	*unknown*		HQ946716
*Niveas agassizi*	Paratype	Kenya	County of Kwale	Mwabungu	08/19/00	David Agassiz	unassigned	*unknown*	female genitalia 137597	
*Niveas agassizi*	Paratype	Kenya	County of Kwale	Mwabungu	08/19/00	David Agassiz	unassigned	*unknown*	female genitalia JR2013-02	
*Niveas agassizi*	Paratype	Kenya	County of Kwale	Mwabungu	08/19/00	David Agassiz	unassigned	*unknown*	wing JR2013-03	
*Niveas agassizi*	Paratype	Kenya	County of Kwale	Mwabungu	08/20/00	David Agassiz	unassigned	*unknown*	female genitalia JR2013-01	

Field sampling and rearing protocols for the PNG material are detailed in Miller et al. (2003; 2013), Craft et al. (2010), and Novotny et al. (2010). The Plant List website (2010) was used for host plant names. Latitude, longitude, and altitude data for the collecting localities is in Table 2.

**Table 2.** Locality information.

Locality	m.a.s.l.	latitude, longitude
Baitabag village & Kau Wildlife Area, near Madang, Madang Province, PNG	50	5°08'S, 145°46'E
Mis, Madang Province, PNG	50	5°11'S, 145°47'E
Ohu Conservation Area, Ohu village near Gum river, Madang Province, PNG	100	5°13'S, 145°41'E
Pau, Madang Province, PNG	0	5°08'S, 145°46'E
Reinduk, Madang Province, PNG	225	5°39'S, 145°24'E
Riwo, Madang Province, PNG	0	5°09'S, 145°48'E
Tab Island, Madang Province, PNG	0	5°10.6'S, 145°52.6'E
Wanang village, Madang Province, PNG	115	5°13.9'S, 145°10.9'E
Mwabungu, County of Kwale, Kenya	0	4°20.3'S, 39°37'E

The molecular phylogeny dataset included three outgroups and 40 species of ingroup taxa, including two individuals each of *Niveas kone* and *Niveas agassizi* totaling 45 terminal units. We analyzed data from eight genes: COI (mitochondrial), CAD, EF1*α*, GAPDH, IDH, MDH, RpS5, and wingless (all nuclear) (Wahlberg and Wheat 2008). The final alignment was 6187 base pairs long. Molecular sequences for all taxa except *Niveas kone* and *Niveas agassizi* are from Rota (2011) and Rota and Wahlberg (2012), and their GenBank accession numbers can be found there. For the specimens of *Niveas kone* (660733) and *Niveas agassizi* (Ch_JR44_1), DNA extraction was done from whole abdomens, which were later used for dissection of genitalia. Because the DNA amplification methods described by Wahlberg and Wheat (2008) did not work for obtaining sequences of nuclear genes from these specimens, suggesting that their DNA was too degraded for the standard approach, we used newly-designed primers (Niklas Wahlberg, unpublished) (Table 3) to amplify short fragments of the nuclear genes (see Table 4 for total number of base pairs for each gene fragment amplified and the GenBank accession numbers for fragments longer than 200 base pairs). For sequence storage and manipulation we used the VoSeq application (Peña and Malm 2012). The nexus file with the alignment is available from the Figshare Digital Repository: http://dx.doi.org/10.6084/m9.figshare.811841

**Table 3.** Primers.

COI-1F	GGTCAACAAATCATAAAGATATTGG
COI-1R	GGwGCyCCTARtATtAaaGGWAYTA
EF-1F	CACATYAACATTGTCGTSATYGG
EF-1R	TrScgGTYTCGAAcTTCCA
EF-2F	GAgCGtGARCGTgGTAT
EF-2R	rGCtTCgAAcTCACCRGTA
EF-3F	TcAAgAACATGATcACyGG
EF-3R	GARGAyACTTCcTTcTTgA
EF-7F	CAAYGTtGGtTTCAACGT
EF-8R	ACAGCVACKGTYTGYCTCATRTC
GAPDH-1F	aargctggrgctgaatatgt
GAPDH-1R	AAGTTgTCaTGgATRACcTT
GAPDH-2F	gTcaTcTCyAAtGCyTCyTG
GAPDH-2R	TaACtTTgCCrACaGCYTT
GAPDH-3F	GtGCccarCARAACATcAT
GAPDH-3R	tcaGCgGCtTCCTTrACcT
IDH-1F	GGWGAYGARATGACNAGRATHATHTGG
IDH-1R	GGactcTTCCACATtTtYTT
MDH-1F	GAYATNGCNCCNATGATGGGNGT
MDH-1R	TCYTTrCGrGCaACYTTRTC
RpS5-1F	atggcngargaraaytggaayga
RpS5-1R	TTgTGwGCRTAcCtrCCrGC

**Table 4.** GenBank accession numbers and the number of base pairs for each gene fragment.

	*Niveas agassizi* (730793)	*Niveas agassizi* (Ch_JR44_1)	*Niveas kone* (730509)	*Niveas kone* (660733)
COI	**HQ946716**	-	**HQ946550**	**KF646130**
	609 bp	176 bp	658 bp	610 bp
EF1*α*	-	**KF646128, KF646129**	-	**KF646131, KF646132**
	-	550 bp	-	706 bp
GAPDH	-	-	-	**KF646133**
	-	136 bp	-	430 bp
IDH	-	135 bp	-	-
MDH	-	190 bp	-	-
RpS5	-	155 bp	-	108 bp

Both maximum likelihood (ML) and Bayesian phylogenetic analyses were performed. ML analysis of unpartitioned data was conducted using RAxML blackbox available online (Stamatakis et al. 2008) with the GTR+G model and 100 bootstraps. Bayesian analysis of data partitioned using the program TIGER (Cummins and McInerney 2011) as described in Rota and Wahlberg (2012) was carried out in MrBayes v. 3.2 (Ronquist et al. 2012) for 10 million generations with one cold and three heated chains, sampling trees every 1000 generations. The analyses were run on the freely available Bioportal server (University of Oslo, Norway). The convergence was assessed by examining plots of log likelihoods and all model parameters using Tracer v.1.5 (Rambaut and Drummond 2007), as well as potential scale reduction factors and split frequencies, both reported by MrBayes. Branch support is expressed as Bayesian posterior probability (PP) and maximum likelihood bootstraps (ML BS).

DNA barcode sequences (COI) for *Niveas kone* (24 specimens) and *Niveas agassizi* (2 specimens) were obtained at the Biodiversity Institute of Ontario, University of Guelph, using their standard methodology (Craft et al. 2010; Hrcek et al. 2011; Wilson 2012). They are deposited in GenBank as accessions listed in Table 1, and their full data including images are in the Barcode of Life Database (http://www.boldsystems.org; see Ratnasingham and Hebert 2007; 2013). These sequences were also analyzed with MrBayes v. 3.2 (unpartitioned dataset, 2 million generations).

## Results

### Taxonomy

#### Niveas

Rota gen. n.

http://zoobank.org/F352952E-0F21-464F-BD1E-278C9A0679C1

http://species-id.net/wiki/Niveas

Figs 1 –9

##### Type species.

*Niveas kone*.

##### Material examined.

See Table 1.

##### Distribution.

Kenya, Papua New Guinea, Solomon Islands.

##### Diagnosis.

*Niveas* can be easily distinguished from most genera of choreutids by the wing pattern (Figs 1, 3). Superficially, species of *Niveas* are similar to some species of *Anthophila* and *Choreutis*, but there is no known species in either of the latter two genera with a black terminal band enclosing white spots in the forewing as in *Niveas agassizi* and *Niveas kone*. (Figs 1, 3). Forewing venation with only four radial branches or with R_4_ and R_5_ fused in the basal half is also diagnostic for the genus. Female genitalia with paired concave sclerotizations on A7 sternite are also unique to *Niveas*.

##### Description.

**Figures 5–9.**
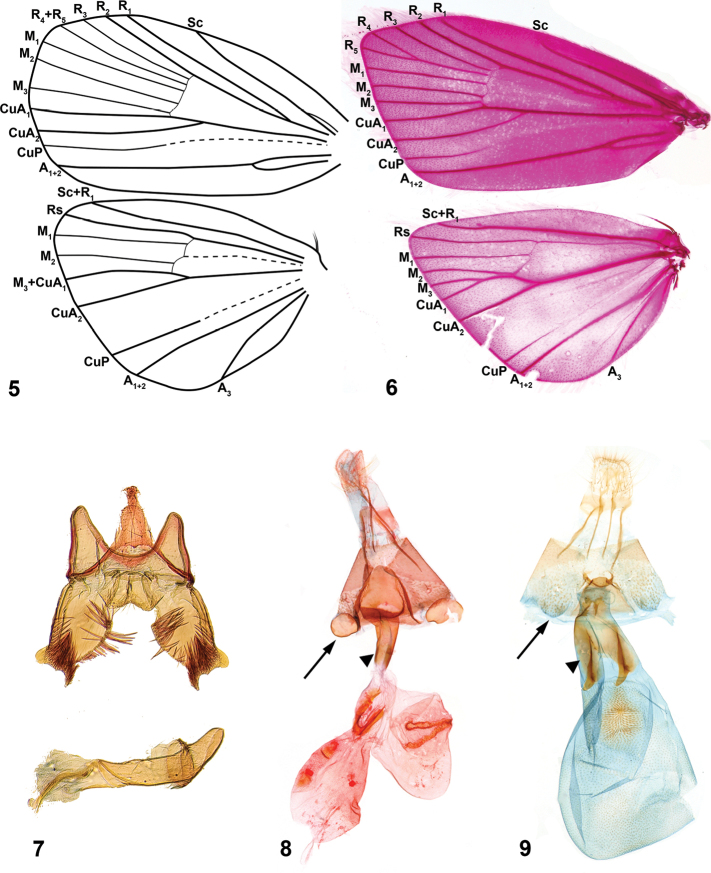
*Niveas kone*: **5** Wing venation **7** Male genitalia **8** Female genitalia. *Niveas agassizi*: **6** Wing venation **9** Female genitalia. (In Figs 8 and 9 arrows point at the A7 sternite sclerotizations, and triangles point at the lateral sclerotizations on the ductus bursae.)

*Head*. Labial palpi with projecting ventral scale tufts (Figs 2, 4). *Wings*. Forewing veins R four-branched in *Niveas kone* (Fig. 5), five-branched in *Niveas agassizi* (Fig. 6), with R_4_ an R_5_ fused in basal 3/5; CuP present at termen for 1/3 to 1/5 wing length, extending as fold further towards base. Hindwing ten-veined, with M_2_ in close proximity to the basally fused M_3_ and CuA_1_ (*Niveas agassizi*) or nine-veined, apparently with M_3_ and CuA_1_ completely fused into a single vein (Figs 5, 6). *Male genitalia*. Tegumen rounded on top, tuba analis extending beyond tegumen; vinculum as inverted trapezoid ventrally emarginate; valva with costal margin straight, ventral margin rounded, ending with a horn-like projection; phallus twice as long as valva (Fig. 7). *Female genitalia*. Apophyses anteriores slightly longer than posteriores; ostium bursae on A7 with a more or less strongly sclerotized antrum; ductus bursae straight, not coiled, with strong lateral sclerotizations; corpus bursae as a single sac (*Niveas agassizi*) or divided into two sacs (*Niveas kone*) with one or more signa. A7 sternite with paired, somewhat rounded, concave sclerotizations proximally, clearly visible in *Niveas kone* (Fig. 8), and slightly less so in *Niveas agassizi* (Fig. 9).

##### Host plants.

Genus *Ficus* (Moraceae).

##### Etymology.

The generic name is derived from Latin *niveum*, meaning snowy, in reference to speckles of white-tipped scales in the wings of the type species; it is not treated as a Latin word and is feminine in gender.

#### Niveas kone

Rota sp. n.

http://zoobank.org/9EA367B0-6B92-48FA-8075-D8D0D0BFA566

http://species-id.net/wiki/Niveas_kone

Figs 1 , 2 , 5 , 7 , 8

##### Material examined.

See Table 1.

##### Material deposited.

The holotype and most paratypes will be retained at USNM, with paratypes distributed to PNG National Agriculture Research Institute (Port Moresby), BMNH, Bishop Museum, Naturalis (Leiden), and CSIRO (Canberra).

##### Distribution.

Papua New Guinea, Solomon Islands.

##### Diagnosis.

*Niveas kone* can be separated from all other known choreutids based on its wing pattern (Fig. 1). Superficially, it is similar to a few species of *Brenthia* Clemens, 1860 and *Litobrenthia* Diakonoff, 1978 owing to its background color, but it lacks iridescent spots along forewing termen, which are always present in those two genera. Both male and female genitalia are very distinct from those of other choreutids (Figs 7, 8).

##### Description.

*Head*. Fig. 2. *Wings*. Fore- and hindwing with brown background color, speckled with white-tipped scales in an irregular pattern; a distinct black band along termen of both wings within which are more or less equidistant white spots (Fig. 1). *Male genitalia*. As for the genus (Fig. 7). *Female genitalia*. Corpus bursae split into two sacs; one sac with a V-shaped signum, the other with two round signa (Fig. 8). *Immature stages*. Fig. 12. See a brief note in text.

##### Host plants.

*Ficus botryocarpa* Miq., *Ficus nodosa* Teijsm. & Binn., *Ficus phaeosyce* K. Schum. & Lauterb., *Ficus pungens* Reinw. ex Blume, *Ficus variegata* Blume, and *Ficus wassa* Roxb. (Moraceae).

##### Etymology.

The species is named after the Finnish Kone Foundation (Koneen Säätiö) in appreciation of their funding of this work. The name is a noun in apposition.

#### Niveas agassizi

Rota sp. n.

http://zoobank.org/7F08322B-C0D2-450C-9DFF-ED9E4FEA5892

http://species-id.net/wiki/Niveas_agassizi

Figs 3 , 4 , 6 , 9

##### Material examined.

See Table 1.

##### Material deposited.

The holotype will be deposited in National Museums of Kenya (Nairobi) (NMK), with paratypes to USNM, BMNH and NMK.

##### Distribution.

Kenya.

##### Diagnosis.

*Niveas agassizi* can be separated from other known choreutids by the wing pattern (Fig. 3). It is superficially similar to some species of *Choreutis*, but the latter usually have forewings with apparent patterning, and this is absent in *Niveas agassizi*. Female genitalia are very distinct from those of other choreutids (Fig. 9).

##### Description.

Male unknown. *Head*. Fig. 4. *Wings*. Forewing bronze-brown with speckled white-tipped scales over most of its surface; distinct dark brown to black band along termen with two small white spots at apex; hindwing light brown (Fig. 3). *Male genitalia*. Unknown. *Female genitalia*. Ductus bursae short and wide, opening into large corpus bursae, with one oval signum (Fig. 9). *Immature stages*. Unknown.

##### Host plants.

Unknown.

##### Etymology.

This species is named after David Agassiz, who collected all the known specimens and made many significant contributions to our knowledge of African micro-moths. The name is a noun in the genitive case.

### Remarks

**Figure 10.**
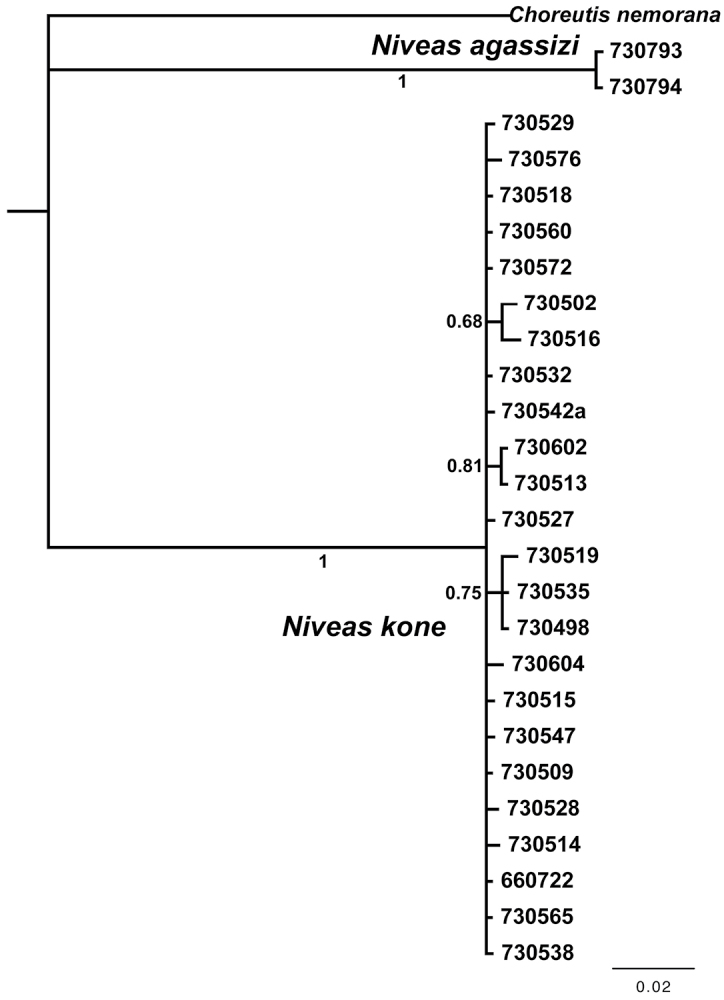
DNA barcode tree from a Bayesian analysis showing low divergence within species and high between species of *Niveas*. Numbers below or next to branches are Bayesian posterior probabilities. Specimen ID numbers are used as labels for the terminal branches.

We obtained 19 full-length barcodes of *Niveas kone*, as well as 5 shorter fragments. These form cluster AAB7478 in the Barcode of Life Database (accessed 29 August 2013), and using the RESL algorithm as implemented there (Ratnasingham and Hebert 2013), the maximum distance between the COI sequences for members of the species is 0.65%, whereas the distance to the nearest cluster (*Niveas agassizi*) is 9.22%. In a Bayesian analysis of the COI sequences, all *Niveas kone* and all *Niveas agassizi* specimens grouped together with the other members of their species with very high branch support (PP=1) (Fig. 10).

**Figure 11.**
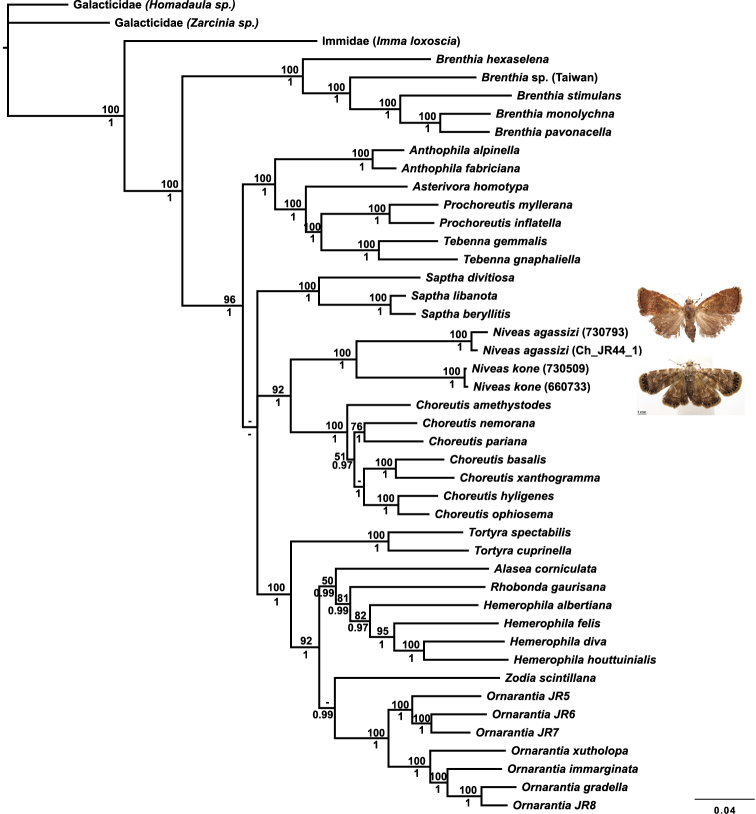
Phylogenetic tree from a Bayesian analysis showing the position of *Niveas* in relation to other choreutid genera. Maximum likelihood (ML) bootstraps are shown above branches, and Bayesian posterior probabilities (PP) are below branches; dashes represent ML bootstraps<50 and PP<0.95.

The placement of *Niveas* in the choreutid generic phylogeny is very strongly supported. *Niveas* clearly belongs within the subfamily Choreutinae (PP=1; ML BS=96), and it appears to be the sister group of *Choreutis* (PP=1.00; ML BS=92) (Fig. 11).

**Figure 12.**
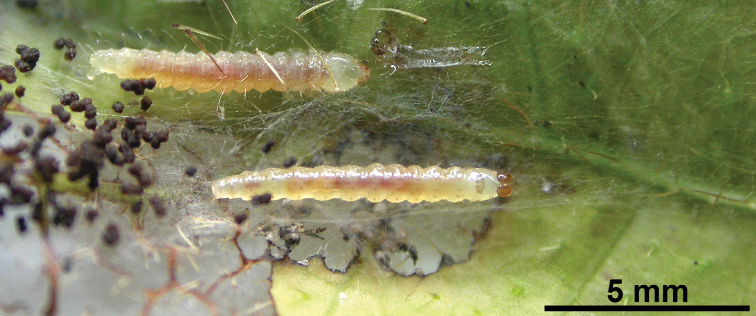
A photograph of the *Niveas kone* larvae made in the field.

Further comments on the biology of *Niveas kone*: Over the years, BRC field teams have encountered larvae identified as *Niveas kone* (as project morphospecies TORT015) 118 times, of which 62 were reared to adults, usually on *Ficus nodosa* and *Ficus variegata*, but also on four other species of *Ficus* (see full host plant list under *Niveas kone* description). Larvae have been found in all months except April and November, and are described by BRC staff as being green-clear-whitish in color, with short white hairs, and one spot on the side of the head (Fig. 12). Larvae of *Niveas kone* share the presence of short hairs with other Choreutinae (Rota 2005), which is unlike Brenthiinae larvae, which possess very long hairs (Rota 2008a). Project field notes indicate that the shelters are distinct from other local Choreutidae in having strong white webbing. BRC has encountered them most commonly in the lowland coastal areas around Madang (city), but also in the coastal mountains behind Madang (up to about 100 m elevation), and at Wanang in the Ramu River Basin (115 m). The species has been recorded in publications (e.g., supplement to Novotny et al. 2010) and online databases as TORT015, misidentified as *Brenthia* sp. Based on locality information provided by Taylor and Maffi (1978: 185, 212), the Solomon Islands specimens are from lowland and foothill localities near Honiara, Guadalcanal; they were collected in light traps.

Taxon descriptions are also organized in tabular format for ease of comparison (see Appendix).

## Discussion

The two species of *Niveas* described herein are superficially quite different, but upon closer examination it becomes apparent that they share a number of morphological features. We consider the following as potential autapomorphies of *Niveas*: fusion or reduction in R veins in the forewing (Figs 5, 6); presence of round, concave sclerotizations on the A7 sternite in females (arrows in Figs 8, 9); strong lateral sclerotizations at the base of the ductus bursae (triangles in Figs 8, 9); and the presence of a terminal black band with white spots in the forewing (arrows in Figs 1, 3). In all other Choreutinae genera there are five fully-separated radial veins in the forewing; the A7 sternite in the female, as well as the base of the ductus bursae, are evenly sclerotized; and if present, a black terminal band in the forewing lacks white spots.

The split between *Niveas kone* and *Niveas agassizi* has presumably happened a long time ago based on the large COI divergence between them and the length of branches in the phylogenetic analysis including the nuclear genes. We considered assigning each species to its own monotypic genus because of their different external appearance, as well as some of the differences in venation and some aspects of female genitalia. It is unfortunate that *Niveas agassizi* is known from females only as perhaps the morphology of the male genitalia would help clarify the status of this species. However, we believe that *Niveas kone* and *Niveas agassizi* being each other’s closest relatives among the currently known species of choreutids is best conveyed by assigning them to a single genus and therefore we opted for this more conservative approach. It is conceivable that other species of *Niveas* that might bridge this gap in both genetic and morphological variation will be discovered in the future. On the other hand, it is also possible that a new genus will need to be erected to accommodate *Niveas agassizi* and its currently unknown relatives.

## Supplementary Material

XML Treatment for Niveas

XML Treatment for Niveas kone

XML Treatment for Niveas agassizi

## Appendix

**Table:** Taxon descriptions organized in tabular format for ease of comparison.

Taxon	*Niveas* Rota, gen. n.	*Niveas kone* Rota, sp. n.	*Niveas agassizi* Rota, sp. n.
**Type species**	*Niveas kone*		
**Material examined**	See Table 1.	See Table 1.	See Table 1.
**Material deposited**		The holotype and most paratypes will be retained at USNM, with paratypes distributed to PNG National Agriculture Research Institute (Port Moresby), BMNH, Bishop Museum, Naturalis (Leiden), and CSIRO (Canberra).	The holotype will be deposited in National Museums of Kenya (Nairobi) (NMK), with paratypes to USNM, BMNH and NMK.
**Distribution**	Kenya, Papua New Guinea, Solomon Islands.	Papua New Guinea, Solomon Islands.	Kenya.
**Diagnosis**	*Niveas* can be easily distinguished from most genera of choreutids by the wing pattern (Figs 1, 3). Superficially, species of *Niveas* are similar to some species of *Anthophila* and *Choreutis*, but there is no known species in either of the latter two genera with a black terminal band enclosing white spots in the forewing as in *Niveas agassizi* and *Niveas kone*. (Figs 1, 3). Forewing venation with only four radial branches or with R_4_ and R_5_ fused in the basal half is also diagnostic for the genus. Female genitalia with paired concave sclerotizations on A7 sternite are also unique to *Niveas*.	*Niveas kone* can be separated from all other known choreutids based on its wing pattern (Fig. 1). Superficially, it is similar to a few species of *Brenthia* and *Litobrenthia* owing to its background color, but it lacks iridescent spots along forewing termen, which are always present in those two genera. Both male and female genitalia are very distinct from those of other choreutids (Figs 7, 8).	*Niveas agassizi* can be separated from other known choreutids by the wing pattern (Fig. 3). It is superficially similar to some species of *Choreutis*, but the latter usually have forewings with apparent patterning, and this is absent in *Niveas agassizi*. Female genitalia are very distinct from those of other choreutids (Fig. 9).
**Description**	Figs 1–9.	Figs 1, 2, 5, 7, 8.	Male unknown. Figs 3, 4, 6, 9.
**Head**	Labial palpi with projecting ventral scale tufts (Figs 2, 4).	Fig. 2.	Fig. 4.
**Wings**	Forewing veins R four-branched in *Niveas kone* (Fig. 5), five-branched in *Niveas agassizi* (Fig. 6), with R_4_ an R_5_ fused in basal 3/5; CuP present at termen for 1/3 to 1/5 wing length, extending as fold further towards base. Hindwing ten-veined, with M_2_ in close proximity to the basally fused M_3_ and CuA_1_ (*Niveas agassizi*) or nine-veined, apparently with M_3_ and CuA_1_ completely fused into a single vein (Figs 5, 6).	Fore- and hindwing with brown background color, speckled with white-tipped scales in an irregular pattern; a distinct black band along termen of both wings within which are more or less equidistant white spots (Fig. 1).	Forewing bronze-brown with speckled white-tipped scales over most of its surface; distinct dark brown to black band along termen with two small white spots at apex; hindwing light brown (Fig. 3).
**Male genitalia**	Tegumen rounded on top, tuba analis extending beyond tegumen; vinculum as inverted trapezoid ventrally emarginate; valva with costal margin straight, ventral margin rounded, ending with a horn-like projection; phallus twice as long as valva (Fig. 7).	As for the genus (Fig. 7).	Unknown.
**Female genitalia**	Apophyses anteriores slightly longer than posteriores; ostium bursae on A7 with a more or less strongly sclerotized antrum; ductus bursae straight, not coiled, with strong lateral sclerotizations; corpus bursae as a single sac (*Niveas agassizi*) or divided into two sacs (*Niveas kone*) with one or more signa. A7 sternite with paired, somewhat rounded, concave sclerotizations proximally, clearly visible in *Niveas kone* (Fig. 8), and slightly less so in *Niveas agassizi* (Fig. 9).	Corpus bursae split into two sacs; one sac with a V-shaped signum, the other with two round signa (Fig. 8).	Ductus bursae short and wide, opening into large corpus bursae, with one oval signum (Fig. 9).
**Immature stages**		Fig. 12. See a brief note in text.	Unknown.
**Host plants**	Genus *Ficus* (Moraceae).	*Ficus botryocarpa* Miq., *Ficus nodosa* Teijsm. & Binn., *Ficus phaeosyce* K. Schum. & Lauterb., *Ficus pungens* Reinw. ex Blume, *Ficus variegata* Blume, and *Ficus wassa* Roxb. (Moraceae).	Unknown.
**Etymology**	The generic name is derived from Latin *niveum*, meaning snowy, in reference to speckles of white-tipped scales in the wings of the type species; it is not treated as a Latin word and is feminine in gender.	The species is named after the Finnish Kone Foundation (Koneen Säätiö) in appreciation of their funding of this work. The name is a noun in apposition.	This species is named after David Agassiz, who collected all the known specimens and made many significant contributions to our knowledge of African micro-moths. The name is a noun in genitive case.
